# A techno-economic perspective on rigid and flexible perovskite solar modules†

**DOI:** 10.1039/d3se00828b

**Published:** 2023-09-26

**Authors:** Lucie McGovern, Erik Christian Garnett, Sjoerd Veenstra, Bob van der Zwaan

**Affiliations:** a University of Amsterdam, Faculty of Science (HIMS, IOP and/or IAS) Amsterdam The Netherlands lucie.mcgovern@free.fr; b AMOLF, Center for Nanophotonics Amsterdam The Netherlands; c TNO Solar Energy, Partner in Solliance Eindhoven The Netherlands sjoerd.veenstra@tno.nl; d TNO Energy Transition Studies Amsterdam The Netherlands; e Johns Hopkins University, School of Advanced International Studies (SAIS) Bologna Italy

## Abstract

Perovskite solar cells have shown considerable developments in the last decade, and commercial applications are drawing closer. In this article, we present a techno-economic study of perovskite PV technologies. We compare published data on manufacturing costs of single-junction perovskite modules and find that they are dependent on the module design (rigid or flexible) and vary from 10 to almost 100 € per m^2^. We calculate the LCOE as a function of module efficiency and stability for a set of four module cost scenarios at 12.5, 25, 50, and 100 € per m^2^. The resulting LCOE varies from 4.3 to 25.5 ct kW^−1^ h^−1^ and shows low potential for immediate competition with crystalline silicon PV in the utility sector. Perovskite PV's competitive advantage lies in both lighter and less rigid modules, and in the development of tandem modules together with silicon. We hence extend the LCOE equation to highlight the benefit of producing flexible low-weight modules by roll-to-roll manufacturing, and modify the LCOE maps to showcase the benefits of tandem modules. Based on learning curve analyses applied to the CAPEX of single-junction and tandem modules, we develop three scenarios for the evolution of the LCOE of perovskite modules from 2025 to 2050. Under the optimistic scenarios, we find that the LCOE could reduce to 2.8 ct kW^−1^ h^−1^ by 2050.

## Abstract 2

What are the conditions for perovskite modules to become competitive against crystalline silicon? We perform a new techno-economic analysis of perovskite PV, where we calculate the LCOE of both rigid and flexible modules, and evaluate cost reductions.

## Graphical abstract

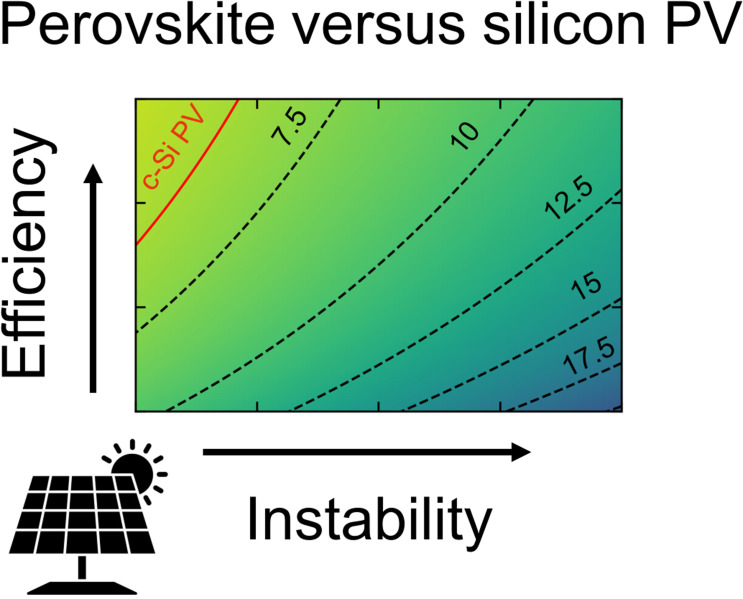

## 1 Introduction

The development of renewable energy is essential to reduce global CO_2_ emissions. With the largest reduction in levelized cost of electricity (LCOE) compared to all other renewable energy sources,^1,2^ solar power is a key player in this endeavour. The photovoltaics (PV) sector is dominated by crystalline silicon (c-Si) PV, which holds 95% of the market.^3^ However, these solar cells are approaching their efficiency limit. To further push the deployment of solar capacity, the focus of attention is shifting towards emerging PV technologies which have the potential to further reduce the overall cost of solar energy and expand its applicability such as on curved surfaces.

Perovskite solar cells have gained a large momentum in this regard, with claims of rapid commercial applications from industrial companies.^4–7^ The appeal of perovskite cells stems from the combination of high lab efficiencies – with a record of 26% power conversion efficiency (PCE) in 2023 (ref. 8) – cheap material costs, and a wide choice in terms of fabrication techniques, including slot die coating,^9^ gravure printing^10^ and thermal evaporation.^11^ Many of these fabrication techniques are compatible with roll-to-roll manufacturing,^12^ which is especially beneficial in terms of production throughput. Moreover, the perovskite material is deposited on flexible and light-weight substrates,^12^ contrary to c-Si wafer processing,^13^ which produces rigid and heavier modules. Roll-to-roll manufacturing therefore extends the scope of applicability of the newly manufactured modules towards emerging and expanding PV sectors, such as indoor PV and building-integrated PV (BIPV).^7,14^

The development of tandem modules – where a perovskite top cell is deposited on top of a silicon bottom cell – represents another promising integration route for perovskite materials in PV. The silicon bottom cell imposes a rigid design to the whole module, but the higher maximal PCE of these perovskite–silicon (per–Si) tandem modules is beneficial for the overall cost of solar energy.^15^

If perovskite materials might therefore be ideal candidates for the next generation of solar modules, both single-junction (SJ) and tandem modules are still under development, with several key unknowns remaining. Indeed, properties such as scalability of small lab cells towards larger-scale modules^16^ and long-term stability^17^ are still active topics of research. Even the general perovskite module architecture, from mesoporous to planar n–i–p,^18^ through inverted p–i–n^19^ and HTM-free^20^ designs, is still a matter of debate;^21^ and tandem modules are not immune to this interrogation, as they can adopt a variety of device architectures, including 2-terminal (2T), 3T or 4T configurations.^22^ These uncertainties in terms of scalability, stability and device architecture make it hard to pinpoint the possible future contributions of these new technologies to the PV sector. With the present work, we aim to answer the following questions: what are the conditions for SJ perovskite and per–Si tandem modules to become competitive with, or even out-compete, c-Si PV in the utility sector? How can roll-to-roll manufacturing contribute to the development of perovskite PV? Which cost reductions can be expected in the future?

To answer our set of questions, we perform a new techno-economic analysis of perovskite PV. We first look at the module cost estimates as established in the literature and highlight the key factors affecting this cost. In a second step, we calculate the LCOE for SJ perovskite modules, considering a wide range of module efficiencies, degradation rates, and manufacturing costs. To take into account the lightness of perovskite modules fabricated by roll-to-roll manufacturing, we propose a modified LCOE equation, with reduced CAPEX. We then extend our LCOE analysis of perovskite materials to tandem modules, and compare the relative benefits between these and the SJ perovskite modules. Finally, we perform learning curve analyses on the CAPEX of both SJ perovskite and per–Si modules, and propose three LCOE scenarios, from conservative to optimistic, for the period 2025–2050.

## 2 Results and discussion

### 2.1. Manufacturing costs for SJ perovskite modules

**Fig. 1:**
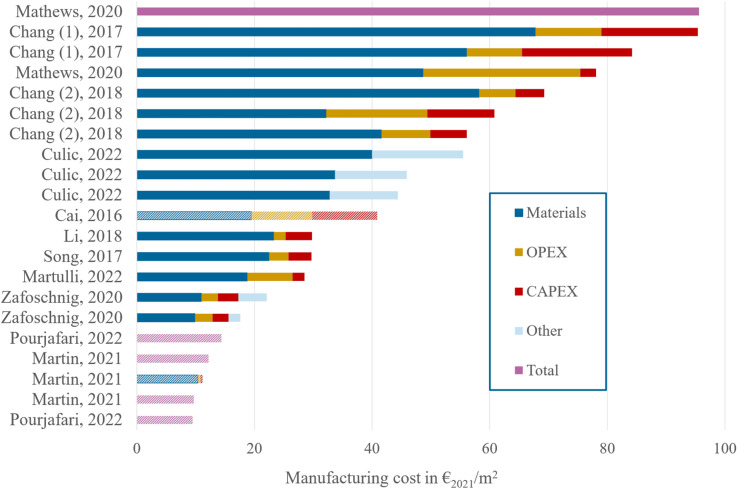
Literature review of manufacturing costs for SJ perovskite solar modules, as calculated in the techno-economic analyses from ref. 23–33. When Balance Of Module (BOM) costs are not included in the calculations,^23,25,31^ the boxes are presented with a hashed shading.

The first focus of our techno-economic analysis is the fabrication cost of the perovskite modules, *i.e.* the total costs for a solar cell manufacturer to produce a perovskite module. This includes the material costs, the operational expenditures (OPEX) for electricity, labour and maintenance requirements of the manufacturing plant, and the capital expenditures (CAPEX) for the facilities and other equipment. While reviewing the literature on the topic,^23–33^ we find that the perovskite manufacturing cost is given in two different units, either in [€ per m^2^] for the module cost per area^24–29,33^ or in [€ per W] for the module cost per power output.^23,30–32^ To allow for a general comparison of all literature values, we keep those reported in [€ per m^2^] as such, and convert those reported in [€ per W] to [€ per m^2^] by using a PCE factor of 18 or 20% – the first for flexible modules and the latter for rigid modules, since the latter are known to yield better performance. These perovskite PCE values are taken directly from the original ref. 30–32 or represent a close approximation thereof.^23^ We then divide the manufacturing costs into the costs of materials, OPEX, CAPEX, “other” and/or “total”. Specifically, our use of the category “other” refers to the costs published by literature references using a different categorisation than the one proposed here, and we use “total” when the costs were not separated into specific categories. We further correct all calculated cost values to account for inflation between the year of their publication and 2021, which we will use as our reference year. Finally, all costs published in dollars are converted to euros. The resulting normalized values for perovskite manufacturing costs are shown in Fig. 1, with a detailed dataset available in Section 1 of the ESI.†

The most striking observation lies in the spread of calculated cost, with an order of magnitude difference between the lowest and the highest estimate, from 10 € per m^2^ (in Pourjafari *et al.*^25^) to almost 100 € per m^2^ (in Mathews *et al.*^30^). A thorough perovskite LCOE calculation should therefore consider the breadth of possible manufacturing costs in order to accurately report the corresponding possible range of LCOE values. We alert for an important caveat in attempts to compare cost values from literature that derive from different methodologies. For instance, the calculations by Cai *et al.*,^23^ Martin *et al.*^31^ and Pourjafari *et al.*^25^ omit the costs of encapsulation, framing, and addition of a junction box to the modules – the so-called balance of module (BOM).^26^ The module costs resulting from these three studies are therefore presented with a hashed shading in Fig. 1, and are not taken into consideration for the remainder of our manufacturing cost analysis.

As shown in Fig. 1, the total manufacturing costs can be decomposed into 3 main categories: material costs, OPEX and CAPEX. We observe that the main contribution in all calculations comes from material costs, which represents at least 50% of the total costs and can increase to up to 85%, while OPEX and CAPEX play a smaller role in the overall costs. Taken together, OPEX, CAPEX and “other” costs amount to a total of 6.5 to 30 € per m^2^; their spread is low in comparison to that of the material costs. A deeper analysis into the composition of material costs reveals that these are driven neither by the perovskite layer nor the electron or hole transport layers, as one might expect, but primarily by the BOM materials, specifically the front substrate, the encapsulation scheme, and the junction box.^26,30,33,34^

**Fig. 2:**
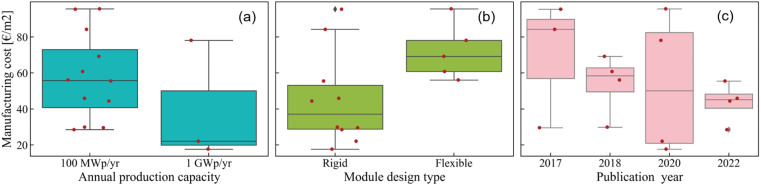
Perovskite module cost as function of (a) annual production capacity of the manufacturing plant, (b) module design type (rigid or flexible) and (c) publication year of the calculation.

The spread in calculated manufacturing costs could be a consequence of the different assumptions made in each of the literature references. The manufacturing capacity, which is a factory's maximum production capability, has, for instance, been shown to be determinant in this regard.^26,30–32^ To study this effect, in Fig. 2a we plot the calculated manufacturing cost as function of the annual production capacity. Most calculations are made with a 100 MWp per year capacity assumption,^24,26–28,30,32,33^ with only a few reports using 1 GWp per year capacity assumption or higher.^29,30^ We find that the median cost is close to 55 € per m^2^ for a 100 MWp per year production capacity and decreases to 22 € per m^2^ with a 1 GWp per year production capacity. This is a reflection of two combined effects: a productivity increase and an economy-of-scale. When the production throughput of a plant increases, if the CAPEX and OPEX remain equivalent, the plant becomes more productive and the cost per module is reduced. Regarding the economy-of-scale, the costs of materials are typically observed to be dependent on the quantities purchased, that is material costs are a function of the production capacity. This phenomenon is explicitly accounted for in some of the calculations in our literature review.^30–32^ Overall, the manufacturing cost thus decreases with a higher production capacity, which makes this factor a key leverage for reducing manufacturing costs.

No techno-economic study so far has looked specifically at a comparison between rigid and flexible modules, *i.e.* the difference between modules prepared on glass substrates and those prepared on flexible substrates, such as in roll-to-roll manufacturing. To study whether this design feature of the module has an effect on the perovskite manufacturing cost, in Fig. 2b we plot the manufacturing cost as function of the module design type. We find that the manufacturing cost typically increases by a factor 75% for flexible modules in comparison to rigid modules, with 70 € per m^2^*versus* 40 € per m^2^ for the latter. This result is not or hardly impacted by the production capacity of the modules (at least not within the production capacity assumptions considered here), since 80% of the datapoints for both rigid and flexible modules refer to an annual production capacity of 100 MWp per year. Our findings could, however, be influenced by the publication year of the study, as we observe a slightly higher fraction of flexible module calculations being older than the rigid module calculations (60% *versus* 40%). Later publication years indeed lead to somewhat lower estimates for the manufacturing cost, as shown in Fig. 2c. However, the trend observed here cannot be explained solely by this effect, and we speculate that the additional costs for the flexible foil and the encapsulation scheme are among the main drivers for the resulting cost difference. Our literature review thus suggests a correlation between module type and overall manufacturing cost for perovskite modules, though more calculations are needed to confirm and refine this hypothesis. Further analysis of the data reported in this literature review (relative to the manufacturing plant location) is provided in Section 2 of the ESI.†

### 2.2. LCOE of perovskite solar modules

The LCOE is a measure of the cost of electricity generation, an essential tool to compare electricity production technologies. Different methods exist for calculating the LCOE, the most common being the annuitizing method, in which the cost is decomposed into a certain number of equivalent annuities, and the discounting method, where each year's contribution is weighed by a discount rate.^35^ Since the annuitizing method relies on a fixed value for annual electricity production^35^ and thus doesn't take into account the degradation of the solar panels over time, we select the discounting method.^34^ In this case, the LCOE is obtained by dividing the discounted sum of costs by the discounted sum of electricity production:^36^
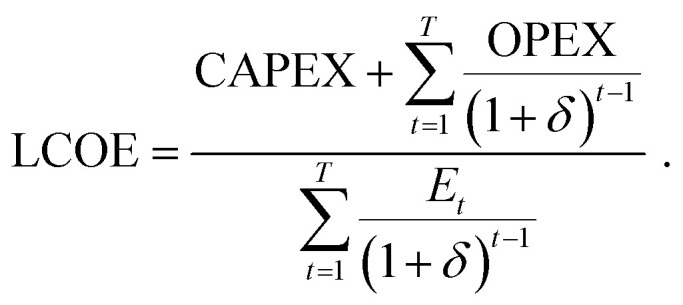
In this equation, CAPEX are the capital expenditures incurred for the installation of a PV solar power plant at time *t* = 0 before the plant starts operating, OPEX the yearly operational expenditures, *δ* the discount rate, *E*_*t*_ the electricity produced by the PV power plant in year *t*, and *T* the total lifetime of the project. The CAPEX can be decomposed into a module contribution and a remaining contribution, called balance of system (BOS): CAPEX = CAPEX_module_ + CAPEX_BOS_. Here, we assume that CAPEX is paid in full during the year of the installation of the system.^37^ The electricity production can be expressed as *E*_*t*_ = PR × Irr × (1 − ADR)^*t*−1^, with PR the performance ratio of the PV modules, Irr the local irradiance, and ADR the annual degradation rate of the PV modules. The PR metric is used to express the actual electricity output from a PV system in comparison to its nameplate capacity, which is defined as the maximum output that this PV system can produce. The PR thus accounts for losses occurring from shading, soiling and DC to AC conversion.^38^

We want to calculate the LCOE for perovskite solar modules in the utility sector, with the goal of comparing it to the LCOE of c-Si modules. As established in the previous section, the range of estimates for the perovskite manufacturing cost remains broad. We thus choose four relevant scenarios for the module cost in order to represent the full scope of module cost variability: 12.5, 25, 50 and 100 € per m^2^. The lower boundary of 12.5 € per m^2^ is less than the realistic current module cost^29^ (at 18 € per m^2^) when all costs are included, but it allows us to visualize the benefits of an ideal low-cost scenario. Apart from the module cost, the LCOE is also dependent on the module efficiency and stability. Since the final specifications of commercial perovskite modules are still unknown, we treat both the efficiency and stability as input parameters, which we vary in order to calculate maps of the LCOE. Regarding the efficiency, the current record for perovskite cells prepared by roll-to-roll manufacturing is 16.7%.^39^ This value has grown thanks to continuous research efforts,^40–42^ but still remains below the record for spin-coated rigid cells, currently at 26%.^43^ These record performances are obtained on a cell level and with aperture areas below 1 cm^2^, while the PCEs of complete modules are lower. In fact, the module PCE decreases with module size: the current record for modules larger than 19 cm^2^ is 21.8%,^44^ which decreases to 17.9%^45^ for cells larger than 800 cm^2^. To cover the full range of potential perovskite performance, we thus choose to vary the module PCE from 10 to 25% in our calculations. For the stability of perovskite modules, we fix the project duration of a solar power plant to 25 years and vary the ADR from values of 0 to 10%. The upper threshold is set at this exceptionally high value of 10% to represent the uncertainty regarding this parameter^34,46^ (see Section 3 of the ESI† for more detail, including an analysis of why a module replacement scheme doesn't provide any LCOE benefit).

To compare perovskite to c-Si PV under the same conditions, we keep the CAPEX_BOS_, OPEX, *δ*, PR and Irr values fixed. The OPEX and CAPEX_BOS_ values for utility-scale PV are taken from IRENA,^47^ as well as the CAPEX_module_ value for c-Si (see details in Section 4 of the ESI†), while the CAPEX_module_ value for perovskite is divided into the four scenarios mentioned above. The *δ* is set to 5% for OECD countries^47^ and the PR is fixed at 85%.^3,37^ We set Irr to 1200 kW h per m^2^ per year, which is the average for global horizontal irradiance in Europe.^48^ Under these conditions, we calculate a LCOE of 6.3 ct kW^−1^ h^−1^ for c-Si PV, assuming 21% PCE^3^ and 0.5% ADR.^49^ This value is larger than the 4.1 ct kW^−1^ h^−1^ average for utility-scale LCOE world-wide,^47^ due to the lower irradiation conditions in Europe compared to the global average irradiation conditions for installed PV.

**Fig. 3:**
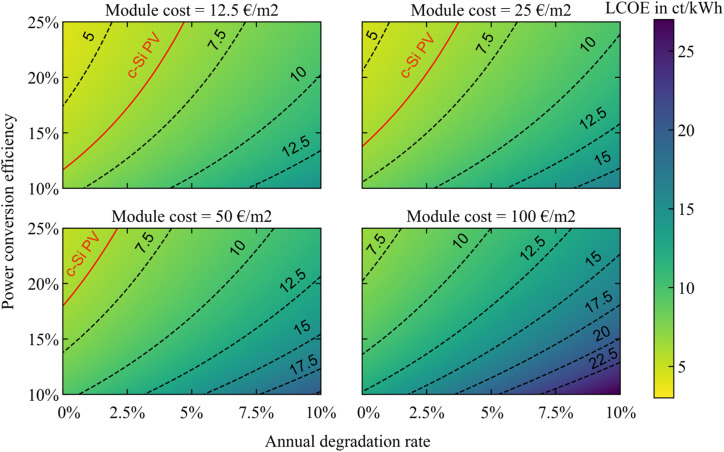
LCOE of SJ perovskite modules as function of their PCE and ADR for manufacturing costs of 12.5, 25, 50 and 100 € per m^2^. Indicated in red is the LCOE for c-Si PV calculated under the same conditions, considering 21% PCE and 0.5% ADR.

The resulting perovskite LCOE maps are shown in Fig. 3. The contours representing 5, 7.5, 10, 12.5, 15, 17.5, 20 and 22.5 ct kW^−1^ h^−1^ are represented by black dotted lines and the LCOE of c-Si PV is shown in red. Our first finding is that all three parameters, namely the module cost, degradation rate, and efficiency, clearly affect the overall LCOE. Only a careful selection of these three parameters will allow for competition against c-Si PV. When the perovskite module cost is as high as 100 € per m^2^, there is no possible competition with c-Si: the LCOE starts from 7 ct kW^−1^ h^−1^ for the high-efficiency, high-stability modules represented in the top left corner of the map, to almost 26 ct kW^−1^ h^−1^ for the modules with low-efficiency and low-stability in the bottom right corner of this map. To be able to compete with c-Si LCOE, the perovskite module cost must be equal to or below 50 € per m^2^, as represented by the c-Si LCOE line (in red) in the matching sub-plots. As expected, with lower module cost, the range of efficiency and stability values over which perovskite modules can compete with c-Si ones expands. With a 1% ADR, the minimal PCE for competition with c-Si decreases from 20% for perovskite modules at 50 € per m^2^ to 16% for modules at 25 € per m^2^ and 13% for modules at 12.5 € per m^2^. Alternatively, perovskite modules with 25% PCE and a 4.5% ADR can still be competitive with c-Si when the module cost is as low as 12.5 € per m^2^, a value which reduces to ADRs of 3.5% and 2% for module costs of 25 € per m^2^ and 50 € per m^2^, respectively. Globally, the minimum achievable LCOE under the present assumptions (*i.e.* for Irr = 1200 kW h per m^2^ per year) is 4.3 ct kW^−1^ h^−1^.

After exploring the combined effects of the variation in module cost, degradation rate, and efficiency in the perovskite LCOE maps, we come to the following conclusion: while competition with c-Si PV for the utility sector is possible when a combination of ambitious module cost, high efficiency and high stability is reached for perovskite modules, this set of conditions remains quite restrictive. Moreover, the relative benefit is rather low, and only represents a 2 ct kW^−1^ h^−1^ difference in the best scenario. Therefore, we do not foresee immediate competition against crystalline silicon PV in the utility sector. This result is in agreement with previous studies.

## 3 The advantage of low-weight modules

Perovskite PV nevertheless offers a set of unique advantages which differentiates it from c-Si PV. With roll-to-roll manufacturing, processing throughputs could reach as high as 50 m min^−1^ for perovskite PV (with 18 m min^−1^ already proven^10^), whereas c-Si PV is mostly manufactured at about 5 m min^−1^ (though progress is still ongoing, with a recent demonstration of a doubling of this processing throughput^50^). The even higher processing throughputs achieved by roll-to-roll manufacturing thus lead to a higher plant production capacity, which in turn results in a reduced module cost, as discussed in the previous section. In other words, the possibility of having modules in the first and second subplots of Fig. 3, at 12.5 and 25 € per m^2^, would be enhanced. Apart from the higher processing throughput, roll-to-roll manufacturing presents two other benefits for newly designed modules, in terms of both flexibility and light-weight properties. Flexible modules can be integrated in a variety of landscapes that were previously unattainable by c-Si PV, while reduced weight of perovskite modules alleviates both urban planning constraints and installation costs. We note that existing light-weight PV from amorphous silicon (a-Si), CIGS or CdTe, all presently show limiting factors to their further growth – either due to theoretical PCE limits for a-Si,^51^ or to the use of rare elements for CIGS and CdTe.^52^

Here, we quantify the benefit of producing low weight perovskite modules by hypothesising that a change in the module weight might express itself as a reduction in the CAPEX_BOS_. Part of the CAPEX_BOS_ is dependent on the area (hence on the PCE of the modules), *e.g.* for racking and mounting, where a higher module PCE will lead to lower BOS costs. The other part is dependent on the capacity of electricity delivered, *e.g.* for the inverter costs, where a larger amount of electricity produced will lead to a larger cost. Overall, the CAPEX thus depends on four parameters: the module cost, the module PCE, and the area-dependent and capacity-dependent terms of CAPEX_BOS_, expressed as CAPEX_BOS_(*a*) and CAPEX_BOS_(*c*):
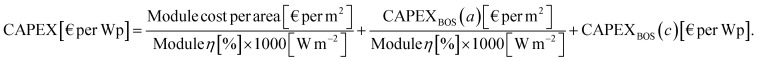


The area-dependent contribution, CAPEX_BOS_(*a*), can be further decomposed into a term which depends only on the module area and not on the module weight, and a term which depends on both the module area and its weight. The table below sums up the different sub-categorisations considered.

**Table:** 

CAPEX_BOS_	Area-dependent CAPEX_BOS_(*a*)	Capacity-dependent CAPEX_BOS_(*c*)
Not impacted by weight	- Electrical installation	- Inverter
	- DC cabling/wiring	- Grid connection
	- Soft costs	- Soft costs
Impacted by weight	- Mounting/racking	
	- Mechanical installation	

**Fig. 4:**
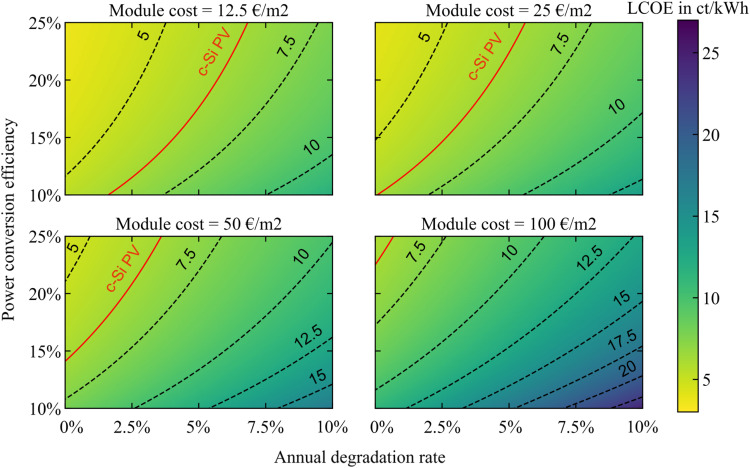
LCOE of low-weight SJ perovskite modules prepared by roll-to-roll deposition, as function of their PCE and ADR for manufacturing costs of 12.5, 25, 50 and 100 € per m^2^. Indicated in red is the LCOE for c-Si PV calculated under the same conditions, considering 21% PCE and 0.5% ADR.

We recalculate the LCOE of perovskite PV for light-weight modules by using a modified LCOE equation, which assumes a factor 10 decrease in the costs related to the weight-dependent term of CAPEX_BOS_(*a*), to illustrate both the potential lower hardware costs in terms of mounting and racking, and the lower mechanical installation costs – for instance with the reduction of labor force physically needed to install the modules. This large decrease factor is used to exemplify the maximal potential benefits for the production of light-weight solar modules within the utility sector (see Section 5 of the ESI† for more detail on this assumption). The results are shown in Fig. 4. In black dotted lines are the contours representing 5, 7.5, 10, 12.5, 15, 17.5, and 20 ct kW^−1^ h^−1^, while LCOE for c-Si PV is highlighted in red.

We obtain a similar set of LCOE maps, in which all three factors – module cost, efficiency and stability – have a role to play in the final perovskite LCOE. We do observe a distinction, however, with respect to the previous case: the LCOE is somewhat reduced and only reaches 23 ct kW^−1^ h^−1^ at most. This also means that the set of conditions for perovskite modules to compete with c-Si PV is less restrictive. Even with a high module cost of 100 € per m^2^, the perovskite modules could be competitive with c-Si based ones if they are 25% efficient and have less than 1% ADR. More realistically, at 25 € per m^2^ and with an ADR of 1%, the perovskite modules would remain competitive against c-Si ones as long as their PCE exceeds 12%. In fact, the sub-plot for modules costing 50 € per m^2^ is similar to the previous one for modules costing 25 € per m^2^ when no light-weight benefit is taken into consideration. In other words, the added value of lightness in these new roll-to-roll deposited modules contributes in reducing the constraint for making cheap modules. Alternatively, with a similar price, the constraints on efficiency and stability of the modules are reduced. Overall, under our modified LCOE equation and for the set of conditions considered here, we find that the LCOE can be reduced down to 3.7 ct kW^−1^ h^−1^, if the perovskite modules are cheap, efficient, and stable. Producing light-weight perovskite modules thus yields a non-negligible impact in terms of the techno-economic benefits of perovskite PV relative to c-Si PV. However, this maximal reduction potential remains limited relative to the 4.3 ct kW^−1^ h^−1^ obtained without any weight considerations, especially considering the advantageous one order of magnitude reduction in weight-dependent CAPEX_BOS_(*a*) used here. In other words, SJ perovskite PV shows limited potential for competition with c-Si PV, even with the additional weight-benefit of using a flexible substrate. We stress that these considerations are valid for the utility sector market, where c-Si PV has been widely established over the last decades, and highlight that perovskite SJ modules might instead unlock their full potential in the residential and industrial sectors, both directly on roof tops and in more intricate designs such as those developed for BIPV.^53^ Indeed, with *e.g.* an estimated 40% of houses in the Netherlands which cannot withhold the weight of traditional c-Si PV modules on their roofs, it becomes clear that light-weight perovskite PV might contribute to new PV markets inaccessible by c-Si PV. Research in SJ perovskite PV should therefore concentrate on these new range of applications which rely specifically on the modules being light and flexible, together with coordinated efforts on both scaling the PCEs obtained on rigid small cells to larger flexible modules and prolonging these modules' outdoors lifetime.

### 3.1. Cost reductions for LCOE of SJ perovskite modules

The LCOE maps shown so far represent the current LCOE values for perovskite modules, if these modules were present on the PV market today. If perovskite modules indeed come to the market, this new technology would continue to mature while it is being deployed worldwide, through the continuation of research and development efforts, optimized manufacturing procedures, as well as economies-of-scale – all of these effects (and others) translating themselves in cost reductions. The learning curve model describes this relationship between the reduction in cost of a produced good and its cumulative capacity.^54^ The cumulative capacity is used as proxy for the experience gained in producing this good. This model has been used for many electricity supply technologies,^55^ including PV.^55–58^ For PV, the global learning rate (LR) was historically found to be between 20 and 25%,^55–57^ defined as a 20–25% reduction in cost for every doubling of cumulative capacity. The LR is dependent on the time period and geographical area of interest, with outlier values as high as *e.g.* 32% for China between 2007 and 2020.^58^ Since perovskite PV are a new class of materials and since the techniques used for their manufacturing are also new, we use the relatively higher-end LR of 25% as our benchmark for the baseline scenario, in order to account for the full scope of accessible learning by doing. We further develop a conservative and an optimistic scenario with LRs of 20 and 30%, respectively. The learning curve analysis is used here directly on CAPEX (and not on LCOE for instance) to showcase the specific impact of each contribution – from modules and from BOS, as explained below – to the overall calculated LCOE value. All three scenarios assume the beginning of SJ perovskite module production by roll-to-roll manufacturing in 2025, with a 1 GWp initial global cumulative installed capacity (CIC). The compound annual growth rate (CAGR), which represents the speed at which cumulative capacity is expected to grow, is set at 20, 25, or 30%^59–61^ for the conservative, baseline, and optimistic scenarios. This leads to global CICs of 6, 7.25 and 8.50 GWp in 2050, respectively in each of these scenarios. The full set of assumptions is reported in Table S2 of the ESI.†

The initial PCEs are set at 12.5, 15, and 17.5% in the conservative, baseline and optimistic scenarios, and the initial costs are fixed to 100, 90, and 70 € per m^2^. Similar to analyses from the international technology roadmap for PV,^62^ we couple the learning curve model described above to a PCE performance advancement of the modules over time. With 0.2, 0.3, and 0.4% per year annual progress rate (APR) within the three respective scenarios, the modules will attain an overall PCE of 17.5, 22.5, and 27.5%^63,64^ in 2050. We couple the faster LR scenario together with higher CAGR, higher initial PCE, and higher PCE APR – and *vice versa* – to represent both the most and least optimistic possibilities, and therefore cover the full range of prospective costs for perovskite PV.

**Fig. 5:**
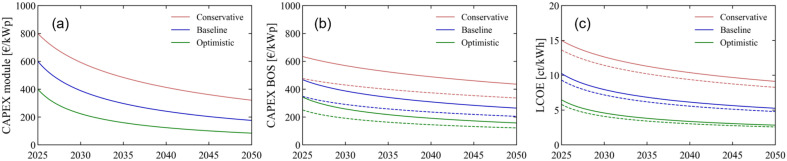
Conservative, baseline, and optimistic scenarios for the time period 2025–2050 for (a) the CAPEX_module_, (b) CAPEX_BOS_, and (c) resulting LCOE of SJ flexible perovskite modules prepared by roll-to-roll manufacturing. The dashed lines in (b) and (c) represent the cost reduction advantage when considering low-weight modules. The LCOE is calculated under average European irradiation conditions.

The resulting CAPEX_module_ scenarios are shown in Fig. 5a, where we observe a significant reduction in cost over time: in the baseline scenario, these decrease from 600 € per kWp in 2025 to 175 € per kWp in 2050; in the optimistic scenario, from 400 to 85 € per kWp; and in the conservative scenario, from 800 to 320 € per kWp. Apart from the module cost, we have seen in the previous sections that the BOS costs can also have a clear impact on the LCOE. The learning curve model has been more often applied to the module costs than to the BOS costs; nonetheless, a recent study found a LR of 11% for the BOS costs in PV.^65^ We thus extend our learning curve analysis to also include the BOS, and develop three CAPEX_BOS_ scenarios, with LRs of 5, 10, and 15%. As seen in Fig. 5b, the BOS cost reductions are smaller compared to the module cost reductions, which is a reflection of the lower LR for BOS. To quantify the potential cost impacts of producing lighter perovskite modules by roll-to-roll manufacturing, we also calculate the effect of a 10-fold reduction in the weight-dependent term of CAPEX_BOS_. The resulting three scenarios are shown in dashed lines. Compared to the scenarios without any weight consideration, there is a relative cost reduction in 2050, from 436 to 337 € per kWp in the conservative scenario, from 265 to 205 € per kWp in the baseline scenario, and from 157 to 122 € per kWp in the optimistic scenario.

As a last step, we use the CAPEX scenarios for both the modules and BOS depicted in Fig. 5a and b as input parameters, fix the *δ* and *E*_*t*_ values, and calculate the corresponding LCOE. Specifically, we maintain *δ* at 5%, PR at 85% and Irr at 1200 kW h per m^2^ per year (as in the LCOE maps shown previously in Fig. 3 and 4) and we vary the OPEX and ADR values as a function of the scenario chosen. The OPEX is set at 10, 15, and 20 € per kWp per year, and the ADR is set to 1%, 2% or 3%; respectively in the optimistic, baseline and conservative cases. The resulting LCOE scenarios are shown in Fig. 5c. In the baseline scenario, the LCOE decreases from 10 to 5.3 ct kW^−1^ h^−1^ between 2025 and 2050; in the optimistic scenario, from 6.5 to 2.8 ct kW^−1^ h^−1^; and in the conservative scenario, from 15 to 9.1 ct kW^−1^ h^−1^. The cost reduction between 2025 and 2050 is thus considerable across all scenarios. We also calculate the three LCOE scenarios when replacing the CAPEX_BOS_ input parameters by their low-weight equivalents: the impact is stronger for the more conservative scenario, and also more noticeable in early years, but relatively small in 2050 in the other two scenarios. In general, the reduction in LCOE is promising for the future of perovskite PV, where both the baseline and optimistic scenarios lead to lower LCOEs than that of c-Si PV today, with 5.3 and 2.8 ct kW^−1^ h^−1^ in 2050. Assuming constant LCOE for c-Si PV, the year in which the LCOE of perovskite modules would become equal to the LCOE of c-Si modules is 2039 for the baseline scenario and 2026 for the optimistic scenario, reduced respectively to 2035 and 2025 if light-weight considerations are taken into account. In other words, modules showing 17.5% PCE together with a cost of 70 € per m^2^, an ADR of 1% and a 10-times decrease in weight-dependent BOS costs, would already be cost-competitive with c-Si PV as early as 2025, with no need for any further LR and PCE improvements. As we do expect c-Si PV to evolve and its LCOE to reduce, we come to the conclusion that it is under the assumptions made in the optimistic scenario that perovskite PV shows the highest opportunity for a competitive advantage against c-Si PV. Finally, we note that the range of LCOE values explored here is calculated under a European irradiation average of 1200 kW h per m^2^ per year. The effects of higher and lower irradiation levels are explored in Section 6 of the ESI.†

### 3.2. Combining perovskite with silicon: the tandem case

So far we have considered SJ perovskite modules, in both their rigid and flexible designs. Another promising application of perovskite PV is in combination with silicon, to create per–Si tandems. These tandem modules can reach higher theoretical efficiencies than their SJ counterparts,^66^ and have already demonstrated a remarkable 33.7% PCE^8^ on a cell level. Here, we extend our LCOE analysis to tandem per–Si modules. Two modifications are necessary to adapt the analysis towards tandem configurations: the PCE parameter is varied from 20 to 40% to represent the potential additional efficiency performance,^67^ and the module cost scenarios are increased by a constant 50 € per m^2^ to represent the additional silicon sub-cell cost. The four module cost scenarios thus become 62.5, 75, 100 and 150 € per m^2^.

**Fig. 6:**
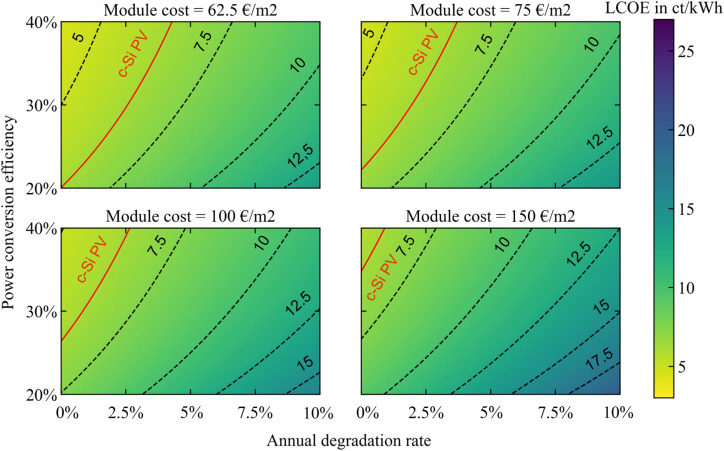
LCOE of per–Si tandem modules as function of their PCE and ADR for manufacturing costs of 62.5, 75, 100 and 150 € per m^2^. Indicated in red is the LCOE for c-Si PV calculated under the same conditions.

The tandem LCOE maps in Fig. 6 show a range of performance values for competition with c-Si PV, across all four modules cost scenarios. To obtain the best cost difference, tandem modules should have a significantly higher PCE than SJ perovskite modules: from 20% PCE for tandem modules at 62.5 and 75 € per m^2^, to 27% for tandem modules at 100 € per m^2^ and even 35% for tandem modules at 150 € per m^2^. The stability requirements are also stronger than in the case of light-weight perovskite modules, with maximum ADRs of 4.4, 3.8, 2.6, and 1.2%, respectively, for the four module costs considered here.

In general, the comparison between per–Si tandem PV and perovskite SJ PV shows similar potential LCOE benefits against c-Si PV in the highest performance regime (with ambitious module cost, high performance and high stability). On the other hand, tandem PV holds a better position than perovskite SJ PV against c-Si PV in the lowest performance regime – the LCOE maximum for tandems is 18 ct kW^−1^ h^−1^ compared to 25.5 and 23 ct kW^−1^ h^−1^ for the classic and light-weight perovskite modules, as shown respectively in Fig. 6, 3 and 4. Nonetheless, the lowest achievable LCOE in Fig. 6 (under the assumptions considered) is at 4.4 ct kW^−1^ h^−1^ – which is almost equivalent to the SJ perovskite modules with no weight consideration from Fig. 3 – and only represents at best less than 2 ct kW^−1^ h^−1^ difference with c-Si PV. Based on the LCOE factor alone, tandem modules might thus not provide a better alternative than c-Si PV in the utility sector. Moreover, tandem modules are more complex to manufacture than SJ modules (from either c-Si or perovskite). However, the LCOE metric does not take into account certain external costs,^68^ such as those associated with land use, which will decrease with the use of more efficient solar modules. Tandem modules (and multi-junction modules in general) might therefore still prove critical in the utility sector, especially in places where land is scarce. We turn to the learning curve analysis to explore the potential for future cost reductions in tandem modules.

### 3.3. Cost reductions for LCOE of tandem modules

**Fig. 7:**
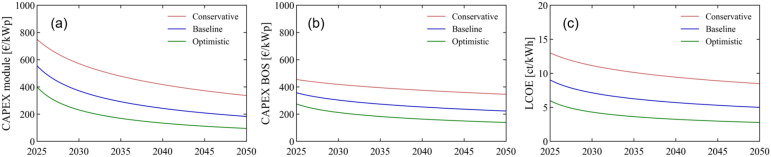
Conservative, baseline, and optimistic scenarios for the time period 2025–2050 for (a) the CAPEX_module_, (b) CAPEX_BOS_, and (c) resulting LOCE of tandem per–Si modules. The LCOE is calculated under average European irradiation conditions.

We perform a similar learning curve analysis for the per–Si tandem modules as for the SJ perovskite modules. The initial CIC in 2025 is set at 1 GWp, and the LRs for CAPEX_module_ and CAPEX_BOS_ are kept at 20, 25, and 30%, and at 5, 10 and 15%,^65^ respectively for the conservative, baseline and optimistic scenarios. The CAGR is varied from 20% in the conservative scenario to 30% in the optimistic scenario, with an intermediate 25% for the baseline scenario, amounting to global CICs of 6, 7.25, and 8.5 GWp in 2050. The initial PCE values of the modules are higher than in the SJ case: here they are set at 20, 25, and 30%, and the same PCE APR is used as in the SJ learning curve analysis, leading to an overall PCE of 25, 30 and 35%^69^ in 2050. The initial module cost is also higher for the tandem modules than their SJ counterparts: here, we fix this cost at 150, 125, and 100 € per m^2^ in 2025. All assumptions are summed up in Table S3,† and the resulting analysis is shown in Fig. 7.

With CAPEX_module_ at 337, 183, and 95 € per kWp in 2050, the scenarios for tandem modules yield similar values, albeit slightly higher, than those obtained for SJ perovskite modules. CAPEX_BOS_ on the other hand are largely reduced, a consequence of the dramatic decrease in area-related costs for these better performing modules. Overall, from 2025 to 2050, CAPEX_BOS_ reduces from 455 to 355 € per kWp in the conservative scenario, from 360 to 225 € per kWp in the baseline scenario, and from 275 to 140 € per kWp in the optimistic scenario. This significant reduction in CAPEX_BOS_ helps to explain the beneficial LCOE trends obtained for tandem modules under the conservative and baseline scenarios, where the LCOE reduces from 13 to 8.5 ct kW^−1^ h^−1^, and from 9 to 5 ct kW^−1^ h^−1^ between 2025 and 2050. The optimistic scenario on the other hand starts at a slightly lower value of 6 ct kW^−1^ h^−1^ (compared to 6.5 ct kW^−1^ h^−1^ for the perovskite SJ modules) but reaches the same final LCOE of 2.8 ct kW^−1^ h^−1^ in 2050. Were the LCOE of c-Si PV to remain constant over this timeline, the year in which the LCOE of tandem modules would become equal to the LCOE of c-Si modules would be either 2035 or 2025, for the baseline and optimistic scenarios respectively – meaning tandem modules at 100 € per m^2^ showing 30% PCE and 1% ADR would already be cost-competitive with c-Si PV as early as 2025. As we assume that the LCOE of c-Si PV will in fact continue to decrease until 2050, the field for possible competition with c-Si PV is mostly reduced to the optimistic scenario conditions. It is therefore clear from this analysis that, firstly, the tandem modules only realise beneficial LCOE values if the assumption of a higher PCE is effectively reached, and, secondly, that it is the set of assumptions behind the optimistic scenario which allow for a competition with c-Si PV in the utility sector. Importantly, the lowest possible value for LCOE – here 2.8 ct kW^−1^ h^−1^ when Irr = 1200 kW h per m^2^ per year (see Fig. S4 and S5 of the ESI† for further analysis under lower and higher Irr conditions) – can be achieved interchangeably by either SJ modules or tandem modules. The future of perovskite PV thus crucially relies on technological developments in terms of efficiency and stability, but also scalability, of each of these types of perovskite technologies.

## 4 Conclusion

In this work, we develop a new techno-economic study for perovskite PV. We start by analyzing the literature regarding the manufacturing cost of SJ perovskite modules, and find a 10-times difference between the lowest and highest estimate. The module cost seems correlated to the type of module (rigid or flexible) and the manufacturing capacity. We then calculate the LCOE for perovskite SJ modules, considering a wide range of manufacturing cost, efficiency, and stability values. We find that all of these parameters matter, and that competition with c-Si PV remains challenging in this initial one-to-one comparison exercise. Direct competition against c-Si PV in the utility sector is therefore not easily foreseen. However, perovskite PV presents a unique advantage over c-Si PV in terms of module fabrication, with the possibility of producing modules through roll-to-roll manufacturing. With this context in mind, we modify the LCOE equation to take into account the low-weight advantage of producing perovskite modules on flexible substrates, as can be realized with roll-to-roll manufacturing. In this case, the LCOE does decrease as expected, but only moderately, making low-weight flexible SJ perovskite modules only mildly more interesting for competition against c-Si PV in the utility sector than the rigid SJ perovskite modules. More importantly, flexible perovskite PV has the potential to reach new PV market sectors previously unachievable with c-Si PV. Our results thus still suggest a strong incentive for the production of light-weight perovskite modules by roll-to-roll manufacturing. Another promising avenue for perovskite PV lies in their integration with silicon to form per–Si tandem modules. We extend our LCOE analysis to such tandem modules, and find a mild reduction in LCOE, similar to that obtained by the SJ modules without any weight consideration. Based on LCOE alone, tandem modules thus also show limited interest for competition with c-Si PV in the utility sector. Their sustained interest lies instead in the reduction of costs external to LCOE, specifically land costs. In all cases, the performance requirements for competition with c-Si PV are stringent, and the modules should combine ambitious PCE on a module level, together with low degradation and low cost. By applying a learning curve analysis coupled to a PCE performance enhancement, we develop three cost reduction scenarios for the LCOE of SJ perovskite modules, from conservative to optimistic, for the time period 2025–2050, and find an LCOE between 2.8 and 9.1 ct kW^−1^ h^−1^ in 2050 (when considering Irr = 1200 kW h per m^2^ per year). An equivalent analysis is performed for tandem modules, which yields LCOEs between 2.8 and 9.5 ct kW^−1^ h^−1^ in 2050. Both types of modules thus yield a similarly low LCOE in 2050, suggesting further research efforts towards efficiency, stability, scalability and cost for both SJ perovskite modules and tandem modules. Crucially, the outcome of these research efforts will determine the identity of the next generation of PV modules, while more fundamental research will help in designing and improving their future successors, from perovskite – perovskite tandem modules to complex multijunction modules with three, four or more junctions.

## Author contributions

Conceptualization, B.v.d.Z., E. G., and S.V.; methodology, investigation and formal analysis, L.M.; writing – original draft, L.M.; writing – review and editing, L.M., E. G., S.V., and B.v.d.Z.; supervision, B.v.d.Z.

## Conflicts of interest

There are no conflicts of interest to declare.

## Supplementary Material

SE-007-D3SE00828B-s001
