# Supplementary material for: A techno-economic perspective on rigid and flexible perovskite solar modules
Source: Sustain Energy Fuels. 2023 Sep 26;7(21):5259–70. doi: 10.1039/d3se00828b (PMC10596340; doi:10.1039/d3se00828b)
Supplement: SE-007-D3SE00828B-s001 [file SE-007-D3SE00828B-s001.pdf]

Electronic Supplementary Material (ESI) for Sustainable Energy & Fuels.
This journal is © The Royal Society of Chemistry 2023

Electronic Supporting Information to

A Techno-Economic Perspective on Rigid and Flexible Perovskite Solar Modules

Lucie McGovern[1,2,*], Erik C. Garnett[1,2], Sjoerd Veenstra[3], and Bob van der Zwaan[1,4,5]

[1] University of Amsterdam, Faculty of Science (HIMS, IOP and/or IAS), Amsterdam, The Netherlands

[2] AMOLF, Center for Nanophotonics, Amsterdam, The Netherlands

[3] TNO Solar Energy, partner in Solliance, Eindhoven, The Netherlands

[4] TNO Energy Transition Studies, Amsterdam, The Netherlands

[5] Johns Hopkins University, School of Advanced International Studies (SAIS), Bologna, Italy

[*] Corresponding author: lucie.mcgovern@free.fr

I. Literature analysis dataset

In Table S1, we present the dataset of perovskite manufacturing costs derived from references [1–11], together with the assumptions made in each of these studies. These results are used to build the manufacturing cost literature review presented in Figure 1 of the main text, as well as the trend comparisons presented in Figure 2. There, most manufacturing costs are included, with the exception of the studies not mentioning BOM costs.

In this literature analysis we consider the manufacturing cost for production of perovskite modules, which is different (and lower) than the perovskite module price, as the price would additionally include a profit margin for the company selling the module.

unit : €2021/m2

	Materials	OPEX	CAPEX	Other	Total	All total [€2021/m2]	Materials / total costs	Comment	Capacity	Design	Publication year	Location
Pourjafari, 2022					9,5	9,5		no BOM		rigid	2022	Mexico
Martin, 2021					9,7	9,7		no BOM	4 GWp/yr	flex	2021	US
Martin, 2021	10,4	0,4	0,4	0,0		11,2	93%	no BOM	2 GWp/yr	flex	2021	US
Martin, 2021					12,2	12,2		no BOM	1 GWp/yr	flex	2021	(US
Pourjafari, 2022					14,4	14,4		no BOM		rigid	2022	Mexico
Zafoschnig, 2020	9,9	3	2,7	2		17,7	56%		1 GWp/yr	rigid	2020	China
Zafoschnig, 2020	11	2,8	3,5	4,8		22,1	50%		1GWp/yr	rigid	2020	China
Martulli, 2022	18,8	7,7	2	0		28,5	66%		100 MWp/yr	rigid	2022	Europe
Song, 2017	22,5	3,3	3,9	0		29,6	76%		100 MWp/yr	rigid	2017	US
Li, 2018	23,3	2	4,5	0		29,9	78%		100 MWp/yr	rigid	2018	China
Cai, 2016	19,5	10,3	11,1	0		40,9	48%	no BOM	100 MWp/yr	rigid	2016	
Culic, 2022	32,8	0	0	11,6		44,4	74%		100 MWp/yr	rigid	2022	Europe
Culic, 2022	33,7	0	0	12,2		45,9	73%		100 MWp/yr	rigid	2022	Europe
Culic, 2022	40	0	0	15,5		55,5	72%		100 MWp/yr	rigid	2022	Europe
Chang (2), 2018	41,6	8,3	6,2	0		56,1	74%		100 MWp/yr	flex	2018	China
Chang (2), 2018	32,2	17,2	11,4	0		60,8	53%		100 MWp/yr	flex	2018	China
Chang (2), 2018	58,2	6,2	4,9	0		69,2	84%		100 MWp/yr	flex	2018	China
Mathews, 2020	48,7	26,7	2,7	0		78,1	62%		1GWp/yr	flex	2020	US
Chang (1), 2017	56,1	9,4	18,7	0		84,2	67%		100 MWp/yr	rigid	2017	China
Chang (1), 2017	67,8	11,2	16,4	0		95,4	71%		100 MWp/yr	rigid	2017	China
Mathews, 2020					95,6	95,6			100 MWp/yr	flex	2020	US

Table S1. Values for the manufacturing cost of SJ perovskite solar modules, as calculated in the techno-economic analyses from the selected references. The assumptions behind each calculation, in terms of plant production capacity, plant location, module design, and publication year of the study, are mentioned together with the reference and manufacturing cost.

II. Module manufacturing cost as function of manufacturing plant location

In Figure 2, we show the impact of multiple criteria on the perovskite manufacturing cost calculation, namely the production capacity of the plant, the design of the module, and the publication year of the study. The location of the manufacturing plant could also impact the perovskite module cost. Traditionally, the assumption was for the factories to be located in either China[2,5–7] or the US[4,8,9], but more recently, works assuming a plant location in Europe have also made their appearance[10,11]. As shown in Figure S1, the manufacturing cost seems to be affected by the plant location, with lower costs in Europe (about 45 €/m^2) than in the US (about 58 €/m^2) and China (about 78 €/m^2). This is surprising if we consider that the American and the Chinese modules each contain about 30% datapoints with a higher production capacity - which should contribute to a lower manufacturing cost - while all European modules rely on the lower 100 MWp/yr assumption. On the other hand, this is probably influenced by the assumption on the module design type, as 2/3 of the American modules and 3/8 of the Chinese modules are also in the flexible design category. Moreover, the calculations made for the European modules are on average younger than those made for the American and Chinese modules, which would again skew the data towards cheaper European modules (see Figure

2.c). The strong correlation between these three variables thus calls for further investigation to disentangle their respective effects.

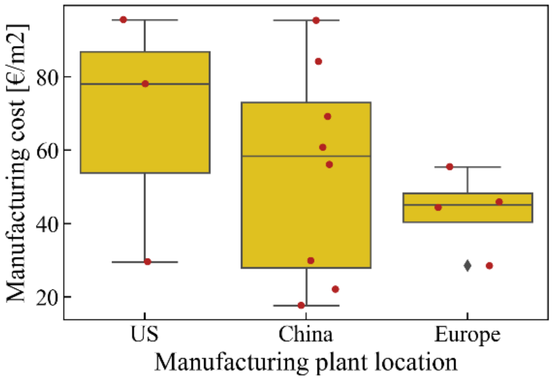

Figure S1. Perovskite module cost as a function of the manufacturing plant location.

III. Module degradation and module replacement

In Figure S2, we show the decreased PCE performance of a given solar module over a time period of 25 years, for ADRs ranging from 0.25 to 10%. The equation for electricity production reported in the main text is $E_t = \mathrm{PR} \times \mathrm{Irr} \times (1-d)^{t-1}$, where the following graph represents the $(1-ADR)^{t-1}$ section. We note that a few references instead use $(1-ADR)^{t}$, which is equivalent to having degraded solar modules in year 1 of their use in the solar power plant. The stability metric can sometimes be expressed by the solar project lifetime rather by the modules' degradation rate[4,12]. In that case, the solar plant's lifetime is usually defined as the time period during which the modules function at a minimum of 80% of their initial performance. Both variables are equivalent and can be retrieved from one another - in Figure S2 we show this intersection between the 80% limit and the perovskite module degradation function.

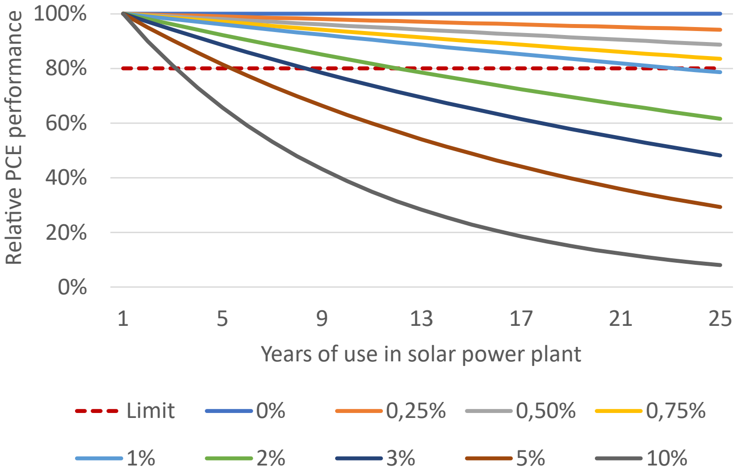

Figure S2. Degradation function of perovskite modules for ADR rates of 0, 0.25, 0.5, 0.75, 1, 2, 3, 5, and 10%.

To evaluate whether module replacement of the poorly-performing modules might be beneficial to the final LCOE, we additionally carry out a module replacement segment to our LCOE calculation. The results are presented in Figures S3 below for the SJ perovskite modules, considering a replacement ceiling set at 50% of the initial PCE performance. For simplicity reasons, we consider only the additional costs when modelling the module replacement contribution to LCOE, and no extra costs incurring from BOS. The modules are replaced as soon as they reach the replacement performance ceiling, and there is no limit to the number of replacements.

In Figure S3 below, we show the effect of applying a 50% replacement ceiling to SJ perovskite modules. Within the range of 0 to 10% considered in our work, this replacement ceiling is equivalent to replacing the solar modules 3 times during the 25 years solar power plant lifetime, i.e. having 4 successive sets of perovskite modules.

We observe that the LCOE increases when considering the module replacement scheme, i.e. that the extra costs occurring from replacing the modules outweigh the benefit from the use of better-performing modules. Within this analysis and for the assumptions mentioned above, it is therefore clear that there is no financial benefit to replace the SJ perovskite modules, and conclude that any solar plant project manager would decide not to invest in module replacement.

We thus conclude that module replacement is not a financial asset for SJ perovskite modules, and pursue our analysis of the LCOE as presented in the main text.

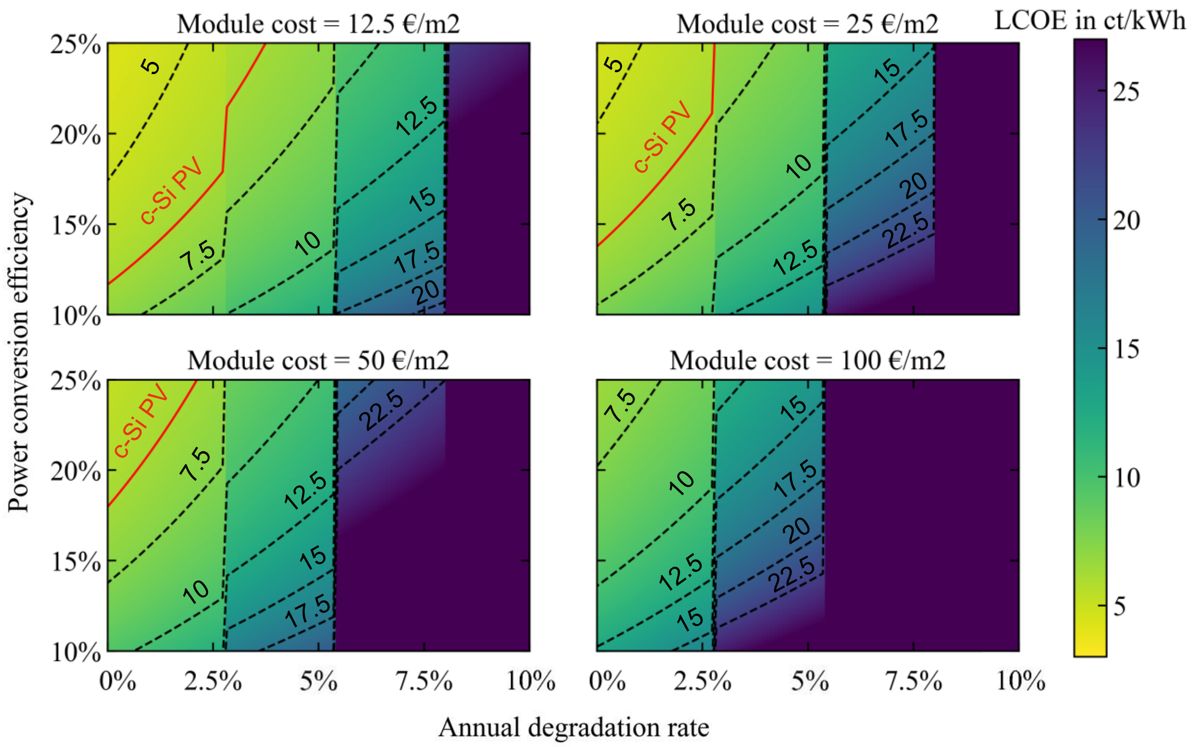

Figure S3. LCOE of SJ perovskite modules as function of their PCE and ADR, for manufacturing costs of 12.5, 25, 50 and 100 €/m^2, considering a replacement scheme when the modules reach 50% PCE relative to their initial performance.

IV. CAPEX and OPEX datasets

In Figures 3, 4 and 6 of the main text, we show LCOE maps of selected types of perovskite PV modules, and include, for comparison purposes, the value for c-Si LCOE. To obtain this map, we first fix the input parameters ($CAPEX_{BOS}$, OPEX, δ, PR and IR) and then resolve the LCOE equation for each set of the variables (PCE, ADR, module cost). The values for both $CAPEX_{BOS}$ and OPEX are taken from IRENA[13]. Specifically, the OPEX for solar PV projects in OECD countries is tabulated at 18.2 USD_{2021}/kWp/yr[13]. We convert it to 15.4 $€_{2021}$/kWp/yr by using the 0.8458 average exchange rate between USD and EUR in 2021[14], and further round it up to a final value of 15 $€_{2021}$/kWp/yr.

For $CAPEX_{BOS}$, we use the same terminology as the IRENA to differentiate these costs into BOS hardware (inverter, racking and mounting, grid connection, cabling / wiring); installation costs (mechanical installation, electrical installation, inspection); and soft costs (margin, financing costs, system design, permitting, incentive application, customer acquisition). We consider the inverter, the grid connection, and half of the soft costs as the capacity-dependent term for the BOS, $CAPEX_{BOS}(c)$. The remainder costs, i.e. racking and mounting, cabling / wiring, all installation costs, and an additional half of the soft costs, are considered as the area-dependent term for the BOS, $CAPEX_{BOS}(a)$.

In the "Renewable Power Generation Costs in 2021" report[13], we find a "breakdown of utility-scale solar PV total installed costs by country", for a set of 36 countries, and using the terminology explained above. From these 36 countries, we select the 21 that are part of the European continent, and average over the relevant sub-sections for $CAPEX_{BOS}(c)$ and $CAPEX_{BOS}(a)$. We find an average of 159 USD_{2021}/kWp for $CAPEX_{BOS}(c)$, equivalent to 134 $€_{2021}$/kWp, and a total average of 320 USD_{2021}/kWp for $CAPEX_{BOS}(a)$, equivalent to 270 $€_{2021}$/kWp. Of these 320 USD_{2021}/kWp for $CAPEX_{BOS}(a)$, 176 USD_{2021}/kWp (equivalent to 149 $€_{2021}$/kWp) are dependent on the area of the modules but not on their weight, and 144 USD_{2021}/kWp (equivalent to 121 $€_{2021}$/kWp) are dependent on both the module area and their weight. The distinction between both categories is presented in the main text.

The $CAPEX_{module}$ value for c-Si n PV is also taken from the "breakdown of utility-scale solar PV total installed costs by country" reported by IRENA[13], using the average of the 21 countries which are part of the European continent. We find 331 USD_{2021}/kWp, equivalent to 280 $€_{2021}$/kWp. If we consider PCE=21%[15], this is also equivalent to 58.8 $€_{2021}/m^2$.

In order to obtain a one-to-one comparison between c-Si PV and perovskite PV in the utility sector, we use the same $CAPEX_{BOS}$ and OPEX values for both c-Si and perovskite modules. This means all costs mentioned above (for electrical installation, grid connection, inverter, etc) are assumed to be equal.

V. Assumption of BOS cost reduction for lighter modules

In our analysis of $CAPEX_{BOS}$ for light-weight modules, we propose a factor 10 reduction of BOS costs for lighter modules, considering lower hardware costs in terms of mounting and racking, and lower mechanical installation costs. This assumption does not cover for potential costs associated to new types of specific supports that might not be at scale yet, neither does it consider additional reinforcement against extreme weather events. On the other hand, our analysis doesn't cover the additional cost reductions associated with lighter package shipments. This cost reduction would push the tendency further towards cheaper costs for lighter modules, and might provide a correction factor if our assumption of lower installation BOS costs turns out to be erroneous.

All in all, it is important to notice that even with the assumption of a substantial 10-times decrease in BOS costs for light-weight modules, our work shows that the competitive advantage obtained

compared to rigid SJ modules remains low for the utility sector, and commercial and residential market sectors would still be the favored segments for light-weight perovskite modules. In other words, whether the light-weight property provides a benefit or a disadvantage against silicon PV in the utility sector, the observed trend of these modules being more fit for BIPV applications remains entirely relevant.

VI. Projected cost reduction scenarios for LCOE under low and high irradiation levels

The assumptions behind the cost reductions scenarios presented in Figures 5 and 7 are reported in the following Tables S2 and S3.

	Conservative	Baseline	Optimistic
LR CAPEX module [%]	20	25	30
LR CAPEX BOS [%]	5	10	15
PCE module [%]	12,5	15	17,5
APR PCE module [%/yr]	0,2	0,3	0,4
CAGR [%]	20	25	30
Initial CIC [GW]	1	1	1
Initial CAPEX module [€/m^2]	100	90	70
Initial CAPEX BOS (a) [€/m^2]	(38 + 22)	(30 + 20)	(22 + 18)
Initial CAPEX BOS (c) [€/kW]	155	135	115
ADR [%/yr]	3	2	1

Table S2. Set of assumptions regarding module CAPEX learning rate, BOS CAPEX learning rate, module PCE, module PCE APR, CAGR, initial CIC, initial module CAPEX, initial BOS CAPEX (area-dependent and capacity-dependent), and ADR for the conservative, baseline and optimistic cost reduction scenarios of SJ perovskite modules, as shown in Figure 5.

	Conservative	Baseline	Optimistic
LR CAPEX module [%]	20	25	30
LR CAPEX BOS [%]	5	10	15
PCE module [%]	20	22,5	25
APR PCE module [%/yr]	0,2	0,3	0,4
CAGR [%]	20	25	30
Initial CIC [GW]	1	1	1
Initial CAPEX module [€/m^2]	150	125	100
Initial CAPEX BOS (a) [€/m^2]	60	50	40
Initial CAPEX BOS (c) [€/kW]	155	135	115
ADR [%/yr]	3	2	1

Table S3. Set of assumptions regarding module CAPEX learning rate, BOS CAPEX learning rate, module PCE, module PCE APR, CAGR, initial CIC, initial module CAPEX, initial BOS CAPEX (area-dependent and capacity-dependent), and ADR for the conservative, baseline and optimistic cost reduction scenarios of per-Si tandem modules, as shown in Figure 7.

In Figure S4 we show the three scenarios (conservative, baseline and optimistic) developed for perovskite SJ LCOE from 2025 to 2050, under both high and low irradiation conditions. The irradiations values chosen here are the GHI's maxima[16]. In the high irradiation case, the LCOE decreases to 1.7 - 4.2 ct/kWh in 2050. With lower irradiation conditions, the LCOE is higher, reaching 4.8 – 14 ct/kWh in 2050. In dashed lines, we additionally represent the reduction in LCOE when the equation is modified to take into account the advantage of producing low-weight modules. In that case, the minimal LCOEs reached in 2050 are 1.3 – 4.1 ct/kWh under maximal irradiation conditions and 3.9 – 12.4 ct/kWh under minimal radiation conditions.

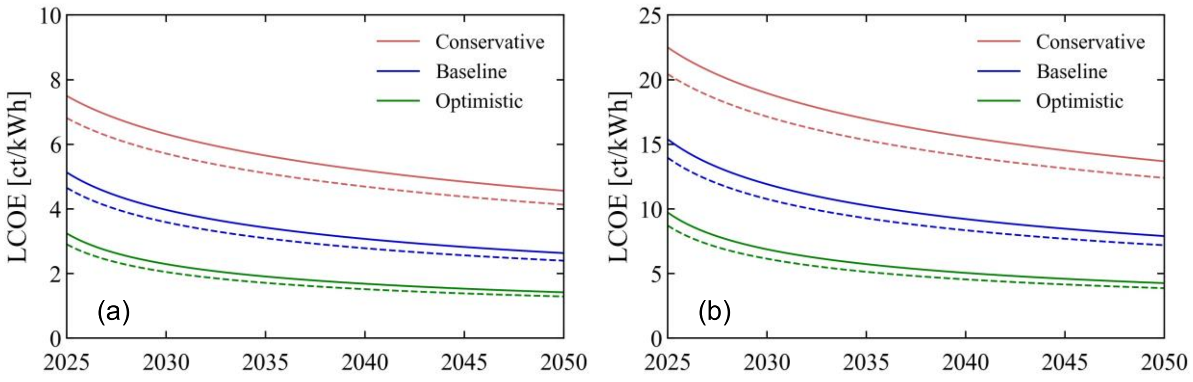

Figure S4. LCOE of flexible perovskite modules prepared by roll-to-roll manufacturing, for the conservative, baseline, and optimistic scenarios covering the time period 2025 – 2050, under (a) high GHI conditions of 2400 kWh/m^2/yr, and (b) low GHI conditions of 800 kWh/m^2/yr. The dashed lines represent the cost reduction advantage when considering low-weight modules.

In Figure S5, we reproduce the previous analysis, this time considering the three scenarios for the LCOE evolution of tandem modules. In the high irradiation case, the LCOE decreases to 1.4 – 4.2 ct/kWh in 2050. With lower irradiation conditions, the LCOE is higher, reaching 4.2 – 12.7 ct/kWh in 2050.

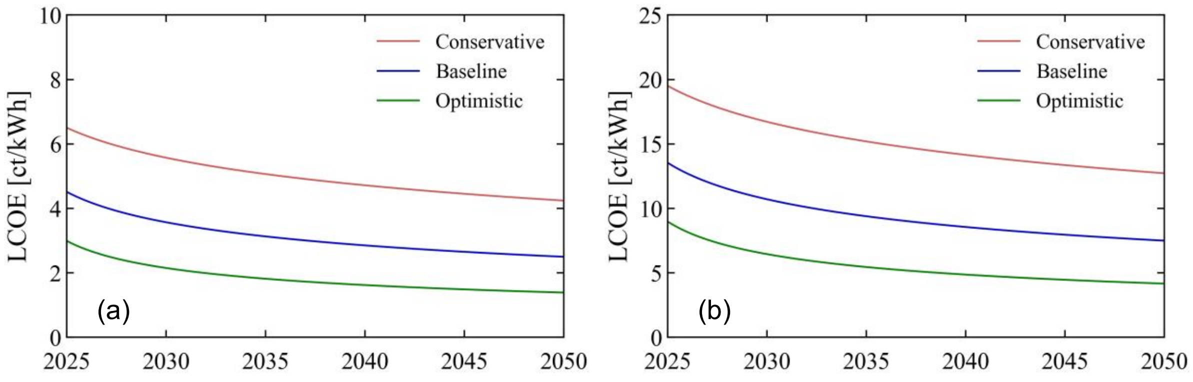

Figure S5. LCOE of tandem per–Si modules, for the conservative, baseline, and optimistic scenarios covering the time period 2025 – 2050, under (a) high GHI conditions of 2400 kWh/m^2/yr, and (b) low GHI conditions of 800 kWh/m^2/yr.

Bibliography

1. Cai, M. *et al.* Cost-Performance Analysis of Perovskite Solar Modules. *Adv. Sci.* **4**, 1600269 (2016).

2. Chang, N. L. *et al.* A manufacturing cost estimation method with uncertainty analysis and its application to perovskite on glass photovoltaic modules. *Prog. Photovoltaics Res. Appl.* **25**, 390–405 (2017).

3. Pourjafari, D. *et al.* Strategies towards Cost Reduction in the Manufacture of Printable Perovskite Solar Modules. *Energies* **15**, 641 (2022).

4. Song, Z. *et al.* A technoeconomic analysis of perovskite solar module manufacturing with low-cost materials and techniques. *Energy Environ. Sci.* **10**, 1297–1305 (2017).

5. Chang, N. L. *et al.* Manufacturing cost and market potential analysis of demonstrated roll-to-roll perovskite photovoltaic cell processes. *Sol. Energy Mater. Sol. Cells* **174**, 314–324 (2018).

6. Li, Z. *et al.* Cost Analysis of Perovskite Tandem Photovoltaics. *Joule* **2**, 1559–1572 (2018).

7. Zafoschnig, L. A., Nold, S. & Goldschmidt, J. C. The Race for Lowest Costs of Electricity Production: Techno-Economic Analysis of Silicon, Perovskite and Tandem Solar Cells. *IEEE J. Photovoltaics* **10**, 1632–1641 (2020).

8. Mathews, I. *et al.* Economically Sustainable Growth of Perovskite Photovoltaics Manufacturing. *Joule* **4**, 822–839 (2020).

9. Martin, B., Amos, D., Brehob, E., van Hest, M. F. A. M. & Druffel, T. Techno-economic analysis of roll-to-roll production of perovskite modules using radiation thermal processes. *Appl. Energy* **307**, 118200 (2022).

10. Čulík, P. *et al.* Design and Cost Analysis of 100 MW Perovskite Solar Panel Manufacturing Process in Different Locations. *ACS Energy Lett.* **7**, 3039–3044 (2022).

11. Martulli, A. *et al.* Towards market commercialization: Lifecycle economic and environmental evaluation of scalable perovskite solar cells. *Prog. Photovoltaics Res. Appl.* (2022) doi:10.1002/PIP.3623.

12. De Bastiani, M., Larini, V., Montecucco, R. & Grancini, G. The levelized cost of electricity from perovskite photovoltaics. *Energy Environ. Sci.* **16**, 421–429 (2023).

13. Renewable Power Generation Costs in 2021. https://www.irena.org/publications/2022/Jul/Renewable-Power-Generation-Costs-in-2021.

14. US Dollar to Euro Spot Exchange Rates for 2021. https://www.exchangerates.org.uk/USD-EUR-spot-exchange-rates-history-2021.html.

15. Fraunhofer ISE. *Photovoltaics Report*. www.ise.fraunhofer.de (2023).

16. Solar resource maps and GIS data for 200+ countries | Solargis. https://solargis.com/maps-and-gis-data/download/france.
